# Editorial: Machine learning for computational neural modeling and data analyses

**DOI:** 10.3389/fncom.2022.978192

**Published:** 2022-07-19

**Authors:** Youhui Zhang, Yiran Chen, Yuanyuan Mi

**Affiliations:** ^1^Tsinghua University, Beijing, China; ^2^Duke University, Durham, NC, United States; ^3^Chongqing University, Chongqing, China

**Keywords:** computational neural modeling, machine learning, brain-inspired computing, neural network simulation, data analysis

In recent years, the development of experimental technology has pushed neuroscience into the era of big data. Facing huge amounts of data, neuroscientists need to model neural networks at multiple levels and preform simulations within different boundaries, which is very challenging. At the same time, AI advances in machine learning (ML) has shown unprecedented capacities in feature extraction and function fitting, with great potential in modeling and data processing. In this Research Topic, we advocate further cooperation between the fields of AI and neuroscience to make more progress. The Research Topic of *Machine Learning for Computational Neural Modeling and Data Analyses*, starting from May 7th, 2021, was organized by Yiran Chen, Yuanyuan Mi, and Youhui Zhang.

Specifically, for neuroscience and computational neuroscience researchers, the requirements contain generating biological neural parameters and synaptic connectivity from experimental data, as well as processing other biophysical models and data. On the other hand, AI can make fundamental contributions to computational neural modeling and data analyses, which can help us gain insight into understanding the inner mechanisms of biological neural network and so on. Anyway, we hope to inspire machine learning researchers to generate efficient methods and tools for processing neuroscience data, so as to change the latter research paradigm.

Therefore, the purpose of this Research Topic is bridging the gap between neuroscience researchers investigating biological models and computer scientists developing brain-inspired methodologies and tools, covering the entire process of modeling and analysis from data cleaning and preparation to modeling, and to simulation and optimization.

Six papers in this Research Topic have been accepted, which involve applying machine learning methods to connectomics, EEG information processing, synaptic tagging and capture (STC), inverse muscle models, neuronal behavior prediction, and more, respectively. The below is an overview and discussion of the accepted articles.

Wang et al. proposed a simple artificial neural network (ANN) to predict spike features of Hodgkin-Huxley-type neurons. Compared with previous work, it can evaluate the informative features of spike, including maximum voltage, minimum voltage, and dropping interval. Different HH-type models were adopted, with firing patterns covering most of the firing behaviors observed in the brain. This work is an intuitive demonstration of the application of ANN in the field of computational neurology.

Another work, proposed by Shi et al., applies deep learning methods used in image to connectomic data analysis. It presents an annotated high resolution image segmentation dataset for cell membrane (U-RISC), which is the largest cell membrane-annotated electron microscopy (EM) dataset with a resolution of 2.18 nm/pixel. The dataset and the deep learning codes used have been publicly available. This open and shared contribution is greatly appreciated and encouraged by the research community.

In the direction of EEG information processing, two papers have been accepted. Bao et al. propose a data augmentation model named for EEG-based emotion recognition, which uses a generative adversarial network to address the lack of training samples. Experimental data shows that the artificial samples can effectively enhance the performance of the EEG-based emotion recognition. It shows high capacities of various methods of deep learning and demonstrates great potential in this field. On the other hand, to solve the problem that high levels of noise limits their usage to strictly noise-controlled environments, Liu et al. present an EEG state-based imputation model based on a recurrent neural network. This method achieves comparable performance to the state-of-the-art methods on the EEG artifact correction task, greatly reducing the amount of manual work required.

Recently, synaptic tagging and capture (STC) theory has become one of the most important theories for the neural mechanism that is about the conversion from rapid change during the early phase of synaptic plasticity into a stable memory trace in the late phase. Ding et al. propose a simplified STC model to takes biological interpretability into account. It can create a wide range of experimental phenomena known in physiological experiments, providing effective learning rules for brain-inspired computing under the premise of ensuring biological authenticity and computational efficiency.

Finally, Nasr et al. propose a machine learning model to resolve the redundancy problem in inverse muscle models, which permits a fast and direct estimation ability instead of iterative solutions for the inverse muscle model. It can be used in various applications, like biomechanical analysis, sports engineering/optimization, functional electrical stimulation control, and so on.

## Author contributions

All authors listed have made a substantial, direct, and intellectual contribution to the work and approved it for publication.

## Conflict of interest

The authors declare that the research was conducted in the absence of any commercial or financial relationships that could be construed as a potential conflict of interest.

The handling editor SW declared a past co-authorship and collaboration with the author YM.

## Publisher's note

All claims expressed in this article are solely those of the authors and do not necessarily represent those of their affiliated organizations, or those of the publisher, the editors and the reviewers. Any product that may be evaluated in this article, or claim that may be made by its manufacturer, is not guaranteed or endorsed by the publisher.

